# Prevalence of Hypertension and Its Clinical and Psychological Factors in Type 2 Diabetes Patients in Ghana: A Secondary Analysis of a Cross-Sectional Study

**DOI:** 10.1155/2024/9286774

**Published:** 2024-11-13

**Authors:** V. Boima, E. Yorke, V. Ganu, L. Twumasi, G. Ekem-Ferguson, D. Dey, I. A. Kretchy, K. Agyabeng, C. C. Mate-Kole

**Affiliations:** ^1^Department of Medicine and Therapeutics University of Ghana Medical School, College of Health Sciences, University of Ghana, Accra, Ghana; ^2^Department of Medicine and Therapeutics, Korle-Bu Teaching Hospital, Accra, Ghana; ^3^Department of Psychology/Center for Ageing Studies, College of Humanities, University of Ghana, Accra, Ghana; ^4^Department of Psychiatry, Korle-Bu Teaching Hospital, Korle-Bu, Accra, Ghana; ^5^Department of Pharmacy Practice and Clinical Pharmacy, School of Pharmacy, College of Health Sciences, University of Ghana, Legon, Accra, Ghana; ^6^Department of Biostatistics, School of Public Health, College of Health Sciences, University of Ghana, Accra, Ghana

**Keywords:** hypertension, prevalence, resilience, risk factors, type 2 diabetes

## Abstract

**Background:** Hypertension (HTN) is common in patients with type 2 diabetes mellitus (T2DM). Patients with both T2DM and HTN have a higher risk of heart disease, kidney disorders, and mortality than those with either HTN or T2DM alone. Patients' psychological well-being plays a significant role in the optimum management of these chronic conditions. This study is aimed at determining the current prevalence of HTN and its related clinical and psychological factors in patients with T2DM.

**Methods:** This cross-sectional study was conducted at the Korle-Bu Teaching Hospital with 156 patients diagnosed with T2DM. A structured questionnaire was used to obtain information on sociodemographic and clinical characteristics. In addition, the following information was obtained from the patients' clinical files: blood pressure, height, weight, waist circumference, serum creatinine, and urine protein. Depression, resilience, and coping skills of the participants were measured using the Brief Symptom Inventory-18, Resilience Scale for Adults, and Brief COPE Inventory, respectively. Data were analyzed using STATA version 18, with a significance level of *p* < 0.05.

**Results:** The median age of respondents was 62.0 (IQR: 51.50, 67.00) years. The majority was female (76.3%). The prevalence of HTN among the patients with T2DM was 79.9% (95% CI: 72.7–85.9). The average body mass index (BMI) of the patients was 28871kg/m^2^ with 34.8% and 36.2% being overweight and obese, respectively. The average HBA1C level was 8.6 ± 2.1 with 71.8% of the patients having poor glycemic control. Increasing age, caregiver, and personal resilience were factors significantly associated with HTN (*p* value of <0.05) among patients with T2DM.

**Conclusion:** The prevalence of HTN among T2DM patients was high; age, caregiver, and personal resilience significantly predicted HTN among T2DM patients. These findings have implications for healthcare providers in implementing strategies to reduce central obesity and incorporating resilience as an important factor in improving treatment outcomes in patients with T2DM.

## 1. Introduction

As the third leading cause of disability-adjusted life years globally, hypertension (HTN) is a significant noncommunicable disease. According to estimates, there will be 1.56 billion adults with HTN worldwide by 2025, up from 972 million in 2000 by 60% with the majority (76%) coming from low-income countries [1]. Individuals with HTN are reported to have a two-fold increased risk of heart disease, seven-fold increased risk of stroke, and four-fold increased risk of congestive heart failure [2].

There is a high prevalence of HTN among patients with diabetes mellitus (DM) in studies from Ethiopia (55.0%) [3], Nigeria (32.1%) [4], and Afghanistan (70.5%) [5]. According to a Kenyan research, 50% of patients with type 2 diabetes mellitus (T2DM) experience HTN [6]. Comparably, another research in Ethiopia indicates that among individuals with T2DM, the prevalence of HTN is 59.5% [7]. Currently, there is very little data on HTN in patients with T2DM in Ghana. Four studies were identified among diabetes mellitus patients: one study of HTN among 100 patients found that 21% had HTN [8], and 37.4% had isolated systolic HTN among 107 patients in another study [9]. The other studies reported uncontrolled HTN among patients with comorbid diabetes and HTN [10, 11].

T2DM and HTN are linked disorders that greatly increase the onset of atherosclerotic cardiovascular disease [12]. HTN is twice as common as T2DM and is associated with 35%–75% of stroke, heart, and kidney disorders [13]. It contributes to diabetic retinopathy, the primary cause of blindness, and nephropathy or chronic kidney disease [12]. Advanced glycosylation end-products accumulate in the arterial wall more quickly in patients with T2DM. This leads to resistance and stiffness in the vessels, ultimately culminating in HTN. Mild-to-moderate hyperglycemia is associated with an increase in sodium retention, which increases total exchangeable sodium and blood pressure (BP) [14]. Another feasible concept is that the overexpression of the renin-angiotensin-aldosterone pathway in T2DM directly affects HTN [15]. Therefore, there is a pathophysiological connection between diabetes and HTN, and understanding this connection will aid both the prevention and creation of early treatment plans.

It is important to pay attention to the coexistence of HTN and T2DM because the risk of death and heart disease or stroke is increased by 44% and 41%, respectively, when both HTN and T2DM occur together, compared with 7% and 9% in individuals with diabetes alone [16]. In addition, psychiatric comorbidities including depression, anxiety, and somatization are frequently linked to chronic diseases such as T2DM and HTN [17]. Among patients with T2DM, concurrent psychiatric disorders are associated with a worse quality of life [18], higher healthcare costs [19], low medication adherence [20], poor glycemic control, more emergency room visits due to diabetic complications [21], and a higher rate of absence at work [22].

As the nation becomes more urbanized, people's diets, physical activities, and the use of tobacco, alcohol, and drugs have changed [23]. As a result, urban dwellers have a greater chance of developing T2DM and HTN. Consequently, to ensure close monitoring of BP and blood sugar levels, patients and healthcare professionals must be aware of the coexistence of HTN and T2DM. The current prevalence of HTN, its awareness and control, and factors linked to HTN in patients with diabetes must be known to ensure adequate control. This study is aimed at ascertaining the prevalence of HTN and its associated risk factors among individuals with T2DM.

## 2. Methods

This study is a subset of a larger cross-sectional study conducted at the Korle-Bu Teaching hospital in Accra, Ghana, and whose detailed methodology has been previously published [24]. Adult patients (≥18 years) with a diagnosis of T2DM or who had been on diabetes medication for at least 6 months in the larger study were identified for analysis in this study. Persons with dementia, neuropsychiatric illness, or a history of trauma were excluded from this study. Written informed consent was obtained from all participants.

A structured questionnaire was used to obtain information on sociodemographic characteristics, including age, gender, religion, educational status, employment status, monthly income, ethnicity, marital status, and socioeconomic status. Standardized questions on household possessions used in the Demographic and Health Surveys in Ghana were used to obtain information on socioeconomic status. The following information was also obtained: clinical parameters, use of pain killers or nonsteroidal anti-inflammatory drugs (NSAIDs), herbal medication, BP readings, height, weight, and waist circumference (WC). Blood samples were taken to the laboratory for the measurement of glycated hemoglobin (HBA1C).

An OMRON HEM-907 electronic sphygmomanometer was used to measure BP. BP was measured three times, at least 5 min apart. The mean of the last two measurements was recorded. HTN was defined as a systolic BP ≥ 140 mmHg and diastolic BP ≥ 90 mmHg [25] or if the patient had already been clinically diagnosed with HTN. Glycemic control was defined as HBA1C < 7% [26].

A stadiometer was used to measure height, with patients wearing no shoes and recorded to the nearest centimeter. The SECA digital weighing scale was used to measure the weight to the nearest 0.5 kg. Body mass index (BMI) was calculated from weight and height. Obesity was defined as BMI30kg/m^2^ and overweight as BMI between 25 and 29.9 kg/m^2^ [27].

WC was measured using a tape measure and recorded to the nearest centimeter. WC of more than 94 cm in men and more than 80 cm in women was considered significant. Hip circumference was with a tape measure and recorded nearest centimeter. This information was used to calculate the waist-to-hip ratio (WHR) [28]. Resilience and coping skills of participants were measured using the revised Adult Resilience Measure (ARM-R) and Africultural Coping Scale Inventory (ACSI), respectively. We adhered to the STROBE guidelines in reporting the results of this observational study (Table S1).

### 2.1. ARM-R

Researchers and practitioners worldwide use the revised ARM-R to assess social and ecological resilience. Its 17 5-point Likert scale questions include “*Not at all*,” “*A little*,” “*Somewhat*,” “*Quite a bit*,” and “*A lot*.” Because of its positive phrasing, scoring involves adding responses. It is categorized into personal resilience involving intrapersonal and interpersonal elements and caregiver resilience involving spouse or family relationships. Cronbach's alpha is 0.87 for the overall resilience subscale, personal, and caregiver resilience subscales [29, 30].

### 2.2. ACSI

The 30-item ACSI examined four culture-specific coping factors: cognitive and emotional debriefing (11 items), collective coping (8 items), spiritual-centered coping (8 items), and ritual-centered coping (3 items). To measure coping mechanisms, ACSI participants used a 4-point Likert-type scale to describe stressful events from the previous week (0 = *not utilized*, 1 = *used a little*, 2 = *used a lot*, and 3 = *used a great deal*). For each subscale, higher scores indicated greater usage of that coping mechanism, determined by adding items. Whole-scale Cronbach's alpha was 0.90 [31].

### 2.3. Statistical Analysis

Data management and analyses were performed using STATA version 18. Summary statistics of categorical variables are reported as frequencies and percentages, whereas those of quantitative variables are reported as mean ± standard deviation if they follow a Gaussian distribution. In cases where the quantitative variable did not follow a Gaussian distribution, the median with its interquartile range was reported. Distribution of continuous variables was exploded with histogram and box-and-whisker plots. Skewness and kurtosis tests for normality were used to formally test normality of continuous variables. The association between categorical variables and HTN status was tested using the chi-square test and Fisher's exact test of independence. The mean and median values between hypertensive and nonhypertensive patients were compared using a two-sample independent *t* test and Wilcoxon rank-sum test. Owing to the problem of overestimation of effect size by the binary logistic regression model, the modified Poisson regression model with robust standard error was used to assess the effects of the patients' characteristics on their HTN status [32]. Prevalence ratio was estimated by exponentiating the coefficient of the modified Poisson model. Variables used in the model were based on literature. All statistical tests were performed at the 5% significance level.

## 3. Results

This analysis comprised 156 Korle-Bu Teaching Hospital T2DM patients aged 18–89 with a median age of 62.0 (IQR: 51.50, 67.00). Females made up 76.3% of patients (*n* = 119) (Table 1). The caregiver and personal resilience scores and median africultural coping scale cognition, spirit, collect, and ritual scores are reported in Table 1.

The prevalence of HTN among T2DM patients was 79.9% (95% CI: 72.7–85.9). Age, employment status, WHR, caregiver resilience score, and coping scale collection score were all associated with high BP (*p* value of <0.05) in the Wilcoxon rank-sum, two-sample independent *t* test, and chi-square test of independence. Patients with HTN had higher median age and collection scores compared to those without HTN. Patients with HTN had significantly higher WHR and caregiver resilience scores compared to those without HTN (Table 1).

The adjusted modified Poisson model revealed significant associations between age, caregiver, personal resilience, and HTN (*p* value of <0.05). With each year of age, HTN prevalence decreased by 1%, along with a 1-point increase in caregiver resilience score. A unit increase in personal resilience increased HTN prevalence by 2% (Table 2).

## 4. Discussion

This study investigated the clinical and psychological predictors of HTN in patients with T2DM at the Korle-Bu Teaching Hospital in Ghana. This study revealed that 79.9% of the patients with T2DM had HTN. This outcome was consistent with previous studies in Libya (85.6%) [33] and Jordan (76%) [34]. Among Ethiopians with T2DM, the pooled prevalence of HTN in a systematic review was 55%. However, the results of this study were higher than those of previous studies in Botswana (61.2%) [35], Nigeria 32.1% [4], Bahrain (38%) [36], and Pakistan (40.45%) [37]. The potential causes of this disparity could include sociodemographic characteristics, study settings, study design, study participants' attendance at the clinic, and lifestyle variations in the study population.

The finding that 71.8% of individuals with T2DM had poor glycemic control aligns with results from another study where 70% had poor glycemic control [38]. Similarly, findings from Uganda (73.52%) [39], Northeast Ethiopia (70.8%) [40], and Saudi Arabia (74.9%) [41] showed high rates of poor glycemic control. However, compared to earlier reports in the USA (69%) [42], India (37.5%) [43], Tanzania (49.8%) [44], and Ghana (51.1%) [10], the degree of inadequate and poor glycemic control status in the current study was greater. The discrepancy between these studies might be due to the setting and index of glycemic control used. For example, our research was conducted in KBTH, a tertiary institution that serves as a referral center for advanced-level healthcare. Therefore, patients with complex and challenging T2DM may be directed to our facility for management. Poor glycemic control is associated with micro- and macrovascular diabetic complications; hence, efforts must be made to achieve control and reduce the rate of these complications.

In this study, more than 70% of individuals with T2DM were either overweight or obese, which is consistent with reports from other studies in Nigeria (83%) [45], Tanzania (85.0%) [46], and Sudan (64.4%); however, a different rate was noted in two Ethiopian studies (23% and 40%) [47]. Similarly, a high prevalence of overweight and obesity was reported in the UK (86%–90%) [48, 49], Australia (53%) [50], and Saudi Arabia (87.5%) among patients with T2DM. Urbanization, globalization, and the adoption of related behaviors such as eating habits and physical inactivity are anticipated to cause a dramatic rise in the incidence of overweight and obesity in sub-Saharan Africa, especially among T2DM patients. The present study found that increasing WHR increased the risk of HTN in patients with diabetes. This is consistent with a Portuguese study that showed that WHR increased the risk of HTN among patients with T2DM [51]. BMI was not a significant predictor of HTN among patients with T2DM in our study, and this may be attributed to the tendency of individuals with T2DM to develop abdominal obesity [52] as measured by WHR in the current study. Consequently, evaluating WHR appears to have potential clinical relevance for determining the risk of HTN in patients with T2DM. Despite the strong correlation between being overweight, obesity, and HTN, individuals with lean body mass have a higher mortality rate from HTN [53]. Our study found a significant association between being underweight and HTN. Education is necessary to enhance nutritional status while ensuring a healthy diet can reduce the potential risk of mortality in underweight patients with HTN.

Positive adaptability throughout adversity is referred to as resilience [54]. Patients with chronic disorders such as T2DM and HTN suffer from a variety of issues, including the long-term nature of the illness, medication burden, frequent hospitalizations, financial restraints, and coexisting conditions. Consequently, adjusting to these circumstances will increase the likelihood that they will manage their sickness successfully and that their treatment will be successful [54]. Resilience and HTN were also linked in this study. Caregiver resilience increased the risk of HTN, whereas personal resilience decreased the risk. Therefore, to help control their HTN, individuals with T2DM must develop personal resilience, as this will help to reduce the development of HTN-related complications in people with T2DM. It is unclear why caregiver resilience increased the risk of HTN among T2DM patients in this study, which requires further investigation. Nonetheless, there could be several reasons for this finding, including the patients' perception of their relationship and the method of caregiving. In subsequent studies, we will investigate the dynamics of caregiving on family involvement in patient care.

Age was another significant factor associated with HTN in the study participants. For each yearly increase in age, the prevalence of HTN increased by 1%. This is in line with a study in Ethiopia, where for each year increase in age, there was a 3% increase in the odds of having HTN in individuals with diabetes [55]. Older people tend to become more sedentary and gain weight, which puts them at a risk for HTN. Age is a nonmodifiable risk factor for HTN, which increases peripheral vascular resistance and is linked to atherosclerotic vascular alterations [56] leading to arterial stiffening and thickening as ageing progresses [57].

Given that this is a secondary cross-sectional study analysis, it was impossible to accurately generalize the findings or establish a causal link between the predictors and HTN. In contrast, this study offers baseline variables linked to HTN in individuals with T2DM. Notably, these findings may be useful in assessing HTN risk among T2DM patients from a clinical standpoint. In addition, the result that caregiver resilience is associated with a higher risk of HTN raises the need to assess the dynamics of caregiver–patient relationships, a topic we will explore in more detail in our upcoming study on family involvement in patient care.

### 4.1. Study Limitations

The study analyzed the data on a subset of patients diagnosed with T2DM among a larger cohort. The authors acknowledge that this approach resulting in a sample size of 156 may not be powered enough for generalizability of our study findings. Thus, study findings should be interpreted with caution. Again, there is a potential gender bias in our study as about two-thirds of our sample population were females. Thus, there is likely a potential reporting bias from the analysis outputs based on gender and therefore interpretation of gender-based results should be done with caution as well.

## 5. Conclusion

To aid the holistic management of HTN with good treatment outcomes, individuals with T2DM should be taught to develop personal resilience, which can help minimize complications associated with HTN. Additionally, the reason why caregiver resilience increased the risk of HTN is not clear and must be explored in the future to determine the dynamics of caregiver–patient relationship in our setting. Future studies on T2DM should be gender-based to produce unbiased findings across genders and also evaluate gender differences to produce gender-specific evidence-based guidance. Understanding this will help in improving patient care especially in our setting where caregiver involvement in patient care is largely practiced.

## Figures and Tables

**Table 1:** The background characteristics and their association with patients with and without hypertension among type 2 diabetic patients.

**Variables**	**Frequency**	**Percentage**	**Hypertension**		**p** ** value**
			**No**	**Yes**	
			**n** ** (%)**	**n** ** (%)**	
Overall (154)			**31 (20.13)**	**123 (79.87)**	
Sex					**0.720**
Male	37	23.72	8 (22.22)	28 (77.78)	
Female	119	76.28	23 (19.49)	95 (80.51)	
Current age: median (LQ, UQ)			51.00 (37.00, 62.00)	63.00 (55.00, 68.00)	**<0.001**
Religion					0.740
Christian	142	91.03	29 (20.71)	111 (79.29)	
Other religions	14	8.97	2 (14.29)	12 (85.71)	
Marital status					0.550
Married/cohabit	89	57.10	16 (18)	71 (82)	
Unmarried	67	42.90	15 (22)	52 (78)	
Educational level					0.370
No formal education	19	12.20	1 (5)	18 (95)	
Primary/JHS	78	50.00	18 (23)	59 (77)	
Senior high school/vocational	40	25.60	8 (21)	31 (79)	
Tertiary	19	12.20	4 (21)	15 (79)	
Current employment status					**0.049**
Employed	77	49.36	20 (26.67)	55 (73.33)	
Unemployed/retired	79	50.64	11 (13.92)	68 (86.08)	
Duration of diabetes (155)					0.009
<5 years	26	16.67	12 (46.15)	14 (53.85)	
5–10 years	26	16.67	17 (65.38)	9 (34.62)	
>10 years	103	66.03	79 (76.70)	24 (23.30)	
BMI (kg/m^2^) (138)					0.500
Mean ± SD	28.79 ± 7.10				
underweight	2	1.45	1 (50.00)	1 (50.00)	
Normal	38	27.78	6 (15.79)	32 (84.21)	
Overweight	48	34.78	12 (25.00)	36 (75.00)	
Obese	50	36.23	9 (18.00)	41 (82.00)	
Waist-to-hip ratio (mean ± SD)	0.90 ± 2.12		0.86 ± 0.07	0.91 ± 0.09	**0.003**
Ever smoked					0.740
Yes	16	10.25	4 (25.00)	12 (75.00)	
No	140	89.74	27 (19.57)	111 (80.43)	
Drinks alcohol (153)					0.180
Yes	23	15.03	8 (34.78)	15 (65.22)	
No	99	64.71	18 (18.56)	79 (81.44)	
Stopped	31	20.26	5 (16.13)	26 (83.87)	
Regular use of pain killers or NSAIDs					0.630
Yes	30	19.23	7 (23.33)	23 (76.67)	
No	126	80.77	24 (19.35)	100 (80.65)	
Use herbal supplements (151)					0.390
Yes	63	41.72	15 (24.19)	47 (75.81)	
No	88	58.28	16 (18.39)	71 (81.61)	
Number of friends you see or hear from at least once a month	3.00 (1.00, 8.00)		4.00 (0.00, 11.00)	3.00 (1.00, 6.00)	0.440
caregiver_arm	30.64 ± 4.83		27.74 ± 6.28	31.33 ± 4.16	**<0.001**
personal_arm	39.20 ± 7.01		39.52 ± 7.45	39.13 ± 6.93	0.790
ACSI cognitive: median (LQ, UQ)	19.50 (14.00, 26.00)		18.00 (10.00, 27.00)	20.00 (14.00, 26.00)	0.400
ACSI spirit: median (LQ, UQ)	15.00 (10.50, 18.00)		14.00 (9.00, 18.00)	15.00 (12.00, 18.00)	0.530
ACSI collect: median (LQ, UQ)	11.00 (7.00, 14.00)		8.00 (6.00, 12.00)	12.00 (8.00, 15.00)	**0.028**
ACSI ritual: median (LQ, UQ)	0.00 (0.00, 0.00)		0.00 (0.00, 0.00)	0.00 (0.00, 0.00)	0.530
Glycemic control					0.460
Mean ± SD	8.59 ± 2.12				
Poor (≥7)	112	71.79	24 (21.62)	87 (78.38)	
Good (<7)	44	28.21	7 (16.28)	36 (83.72)	
Serum creatinine level: median (LQ, UQ)	81.00 (63.00, 95.00)				

*Note:* Bold items refer to items that were statistically significant.

Abbreviations: %, row percentage; ACSI, Africultural Coping Scale Inventory; BMI, body mass index; HBA1C, glycated hemoglobin; JHS, Junior high school; LQ, lower quartile; NSAIDs, nonsteroidal anti-inflammatory drugs; SD, standard deviation; UQ, upper quartile.

**Table 2:** Effect of background, anthropometric, and psychometric characteristics on good glycemic control among patients with diabetes receiving treatment at the Korle-Bu Teaching Hospital.

	**Unadjusted regression model**		**Adjusted regression model**	
	**uPR (95% CI)**	**p** ** value**	**aPR (95% CI)**	**p** ** value**
Age	0.99 (0.99–0.99)	<0.001	0.99 (0.99–1.00)	0.038
Sex				
Male	1		1	
Female	0.93 (0.81–1.06)	0.251	0.96 (0.82–1.12)	0.572
Religion				
Christian	1		1	
Other religions	0.93 (0.77–1.12)	0.427	1.06 (0.89–1.26)	0.491
Marital status				
Married/cohabit	1		1	
Unmarried	1.06 (0.95–1.18)	0.323	1.08 (0.96–1.21)	0.186
Educational level				
No formal education	1		1	
Primary/JHS	1.15 (0.98–1.35)	0.089	1.08 (0.88–1.32)	0.479
Senior high school/vocational	1.17 (0.97–1.40)	0.094	1.05 (0.84–1.32)	0.67
Tertiary	1.05 (0.85–1.29)	0.675	0.98 (0.76–1.26)	0.862
Current employment status				
Employed	1		1	
Unemployed/retired	0.86 (0.77–0.95)	0.005	0.97 (0.84–1.11)	0.644
BMI (kg/m^2^)				
Underweight	1.14 (0.71–1.84)	0.59	1.59 (1.17–2.16)	0.003
Normal	1		1	
Overweight	0.98 (0.84–1.14)	0.81	1.13 (0.98–1.32)	0.100
Obese	0.97 (0.84–1.13)	0.717	1.10 (0.95–1.28)	0.208
Waist-to-hip ratio	0.79 (0.38–1.64)	0.531	0.90 (0.32–2.50)	0.834
Number of friends do you see or hear from at least once a month	1.01 (0.99–1.02)	0.274	1.01 (1.00–1.03)	0.088
Ever smoked				
No	1		1	
Yes	0.96 (0.80–1.14)	0.627	0.95 (0.76–1.17)	0.609
Drinks alcohol				
No	1		1	
Yes	1.08 (0.92–1.27)	0.319	1.03 (0.87–1.20)	0.752
Stopped	1.03 (0.89–1.19)	0.675	1.12 (0.95–1.31)	0.190
Regular use of pain or anti-inflammatory medicines or NSAIDs				
No	1		1	
Yes	1.06 (0.92–1.22)	0.395	1.14 (0.97–1.34)	0.101
Use herbal supplements				
No	1		1	
Yes	1.03 (0.92–1.15)	0.622	0.96 (0.84–1.09)	0.515
caregiver_arm	0.98 (0.97–0.99)	<0.001	0.98 (0.96–0.99)	0.001
personal_arm	1.00 (1.00–1.01)	0.432	1.02 (1.00–1.03)	0.009
ACSI cognitive	1.00 (0.99–1.01)	0.873	1.00 (0.99–1.01)	0.809
ACSI spirit	0.99 (0.98–1.01)	0.266	0.99 (0.97–1.00)	0.068
ACSI collect	0.99 (0.98–1.00)	0.137	1.00 (0.98–1.01)	0.619
ACSI ritual	0.98 (0.94–1.02)	0.298	0.97 (0.93–1.01)	0.121
Glycemic control				
Good	1		1	
Poor	0.98 (0.87–1.11)	0.775	0.92 (0.82–1.03)	0.163
Duration of diabetes				
<5 years	1		1	
5–10 years	0.88 (0.73–1.05)	0.157	0.95 (0.77–1.17)	0.605
>10 years	0.80 (0.70–0.92)	0.002	0.95 (0.81–1.12)	0.528

Abbreviations: ACSI, Africultural Coping Scale Inventory; aPR, adjusted prevalence ratio; BMI, body mass index; CI, confidence interval; JHS, junior high school; NSAIDs, nonsteroidal anti-inflammatory drugs; uPR, unadjusted prevalence ratio.

## Data Availability

The Department of Medicine and Therapeutics at the University of Ghana Medical School will provide the dataset utilized in this study upon request. Please send requests for datasets to B.V. (vboima@ug.edu.gh), the corresponding author.
